# Genome-Wide Binding Map of the HIV-1 Tat Protein to the Human Genome

**DOI:** 10.1371/journal.pone.0026894

**Published:** 2011-11-04

**Authors:** Céline Marban, Trent Su, Roberto Ferrari, Bing Li, Dimitrios Vatakis, Matteo Pellegrini, Jerome A. Zack, Olivier Rohr, Siavash K. Kurdistani

**Affiliations:** 1 Institut de Virologie, Université de Strasbourg, Strasbourg, France; 2 Department of Biological Chemistry, David Geffen School of Medicine, University of California Los Angeles, Los Angeles, California, United States of America; 3 Division of Oral Biology and Medicine, School of Dentistry, University of California Los Angeles, Los Angeles, California, United States of America; 4 Division of Hematology and Oncology, Department of Medicine, David Geffen School of Medicine, University of California Los Angeles, Los Angeles, California, United States of America; 5 Department of Molecular, Cellular, and Developmental Biology, University of California Los Angeles, Los Angeles, California, United States of America; 6 Eli and Edythe Broad Centre of Regenerative Medicine and Stem Cell Research, University of California Los Angeles, Los Angeles, California, United States of America; 7 Institut Universitaire de France (IUF), Paris, France; 8 Department of Pathology and Laboratory Medicine, David Geffen School of Medicine, University of California Los Angeles, Los Angeles, California, United States of America; Beckman Research Institute of the City of Hope, United States of America

## Abstract

The HIV-1 Trans-Activator of Transcription (Tat) protein binds to multiple host cellular factors and greatly enhances the level of transcription of the HIV genome. While Tat's control of viral transcription is well-studied, much less is known about the interaction of Tat with the human genome. Here, we report the genome-wide binding map of Tat to the human genome in Jurkat T cells using chromatin immunoprecipitation combined with next-generation sequencing. Surprisingly, we found that ∼53% of the Tat target regions are within DNA repeat elements, greater than half of which are Alu sequences. The remaining target regions are located in introns and distal intergenic regions; only ∼7% of Tat-bound regions are near transcription start sites (TSS) at gene promoters. Interestingly, Tat binds to promoters of genes that, in Jurkat cells, are bound by the ETS1 transcription factor, the CBP histone acetyltransferase and/or are enriched for histone H3 lysine 4 tri-methylation (H3K4me3) and H3K27me3. Tat binding is associated with genes enriched with functions in T cell biology and immune response. Our data reveal that Tat's interaction with the host genome is more extensive than previously thought, with potentially important implications for the viral life cycle.

## Introduction

After gaining entry into host cell, the HIV-1 genome is reverse-transcribed and the proviral DNA is integrated into the host genome. Subsequently, the HIV-1 provirus is transcribed allowing assembly and release of new viral particles from the infected cell. HIV-1 Tat is essential for efficient viral gene expression and replication [1]. By recruiting the general RNA polymerase II elongation factor P-TEFb to Tat response element (TAR) that forms at the 5′ end of nascent viral transcripts, Tat promotes efficient elongation of viral transcription [2]. Moreover, Tat acetylation by cellular histone acetyltransferases (HATs) such as p300, CBP and PCAF is crucial for its transactivation activity [3]. While the role of Tat in viral gene expression has been well studied, much less is known about the interaction of Tat with the host genome. Previous studies that aimed to define the role of Tat at the host gene promoters found that Tat regulates transcription of the interleukin 6 [4], MHC class I [5], ß2 microglobulin [6] and mannose receptor [7] promoters. Tat also induces host cell apoptosis through association with promoters of PTEN and two PP2A subunits [8]. Therefore, Tat may have roles in regulation of gene expression from the viral as well as the host genome. However, a genome-wide map of Tat interaction with the human genome is still lacking. Such a binding map may reveal additional roles for Tat in creating the proper cellular environment for generating progeny virions.

To generate a genome-wide map of Tat binding to the human genome, we performed chromatin immunoprecipitation combined with next generation sequencing (ChIP-seq) of Tat in Jurkat T cells (Jurkat-Tat). We also utilized microarrays to compare global gene expression changes in Jurkat-Tat versus Jurkat T cells and related the expression differences to histone acetylation changes. We found that the bulk of Tat binding sites are outside the immediate promoter regions of genes. Intriguingly, Tat binds preferentially to specific DNA repetitive elements, especially the Alu repeat elements. Binding of Tat to the promoter regions did not correlate with gene expression. The majority of Tat binding sites at gene promoters in Jurkat-Tat cells are in close proximity with regions bound by the ETS1 transcription factor or CBP in Jurkat T cells. Our data provide the first comprehensive map of Tat binding to the human genome, revealing an unexpected array of target regions.

## Results

### Genome-wide Tat binding locations defined by ChIP-seq

To determine whether Tat binds to specific regions in the host genome, we performed ChIP-seq to map Tat binding sites in Jurkat T cells that stably express Tat under G418 selection [9]. We first validated Tat expression in Jurkat-Tat cells with Western blotting (Figure S1). Subsequently, we sequenced both input and ChIPed Tat-bound DNA using the Illumina GAIIx Sequencer. The obtained sequences were aligned to the human genome (version Hg19) using the Bowtie software [10]. For both input and ChIPed samples, ∼62% of all sequences were uniquely aligned to the human genome (Table 1). We segmented the human genome into 100 bp windows and calculated the ChIPed DNA read counts, which were compared to input DNA read counts in each window. Using the Poisson distribution, we calculated *P*-values for the enrichment of ChIPed reads in each window. Significant peaks were defined as those windows with a *P*-value<10^−4^ and with two neighboring windows at the same significance *P*-value. Based on these criteria, we identified 2074 genomic regions occupied by Tat in Jurkat-Tat T cells.

**Table 1:** ChIP-seq alignment results using Bowtie 0.12.7 (Hg19).

DNA	Total reads	Aligned Reads (%)
Input	42249133	26416727 (62.5)
ChIPed	44360277	27831745 (62.7)

**Figure 1:**
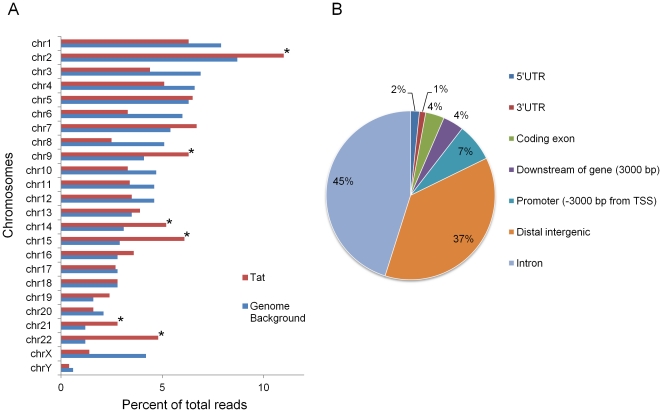
Chromosomal distribution and genomic location of Tat binding sites. (**A**) Enrichment pattern of Tat-bound regions among individual chromosomes is shown as a bar chart. Percent of total Tat-binding sites (red bars) and what would be expected by random chance (blue bars) for each chromosome is shown. The asterisks denotes enrichment *P*-value<10^−4^. (**B**) Distribution of all Tat-binding peaks in relation to gene structure is shown as a pie chart. Intergenic regions are defined as at least 3 kb away from the start and end of any transcript.

Using the cis-regulatory element annotation system (CEAS) software [11] to determine the Tat binding distribution pattern across individual chromosomes, we observed that chromosomes 2, 9, 14, 15, 21 and 22 were significantly (*P*<10^−4^) enriched for Tat binding (Figure 1A). The majority (82%) of Tat binding occurs within introns and intergenic regions in the genome (Figure 1B). Intergenic regions are defined as those regions that are at least 3 kilo bases (kb) away from any known gene. Only ∼7% of Tat binding sites are located within the promoter regions. These data suggest that Tat binding to the genome is non-random, with preferential binding to certain chromosomes and intergenic regions.

### Tat binding loci are enriched within repeat sequences

**Figure 2:**
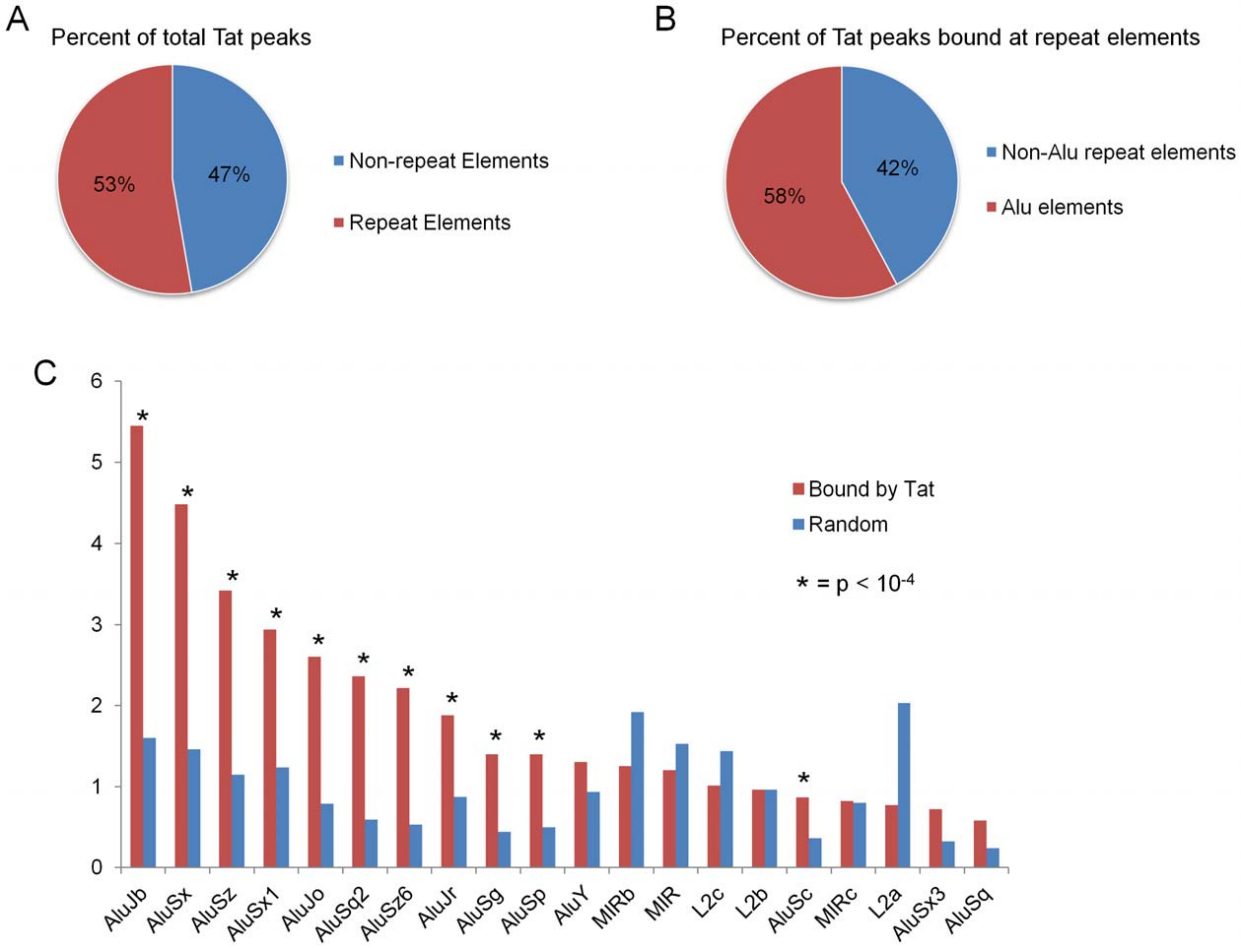
Tat binds mainly to DNA repeat elements. (**A**) Distribution of Tat-binding peaks in repeat versus non-repeat elements is shown as a pie chart. (**B**) Distribution of Tat-binding peaks within repeat elements in Alu versus non-Alu sequences is shown as a pie chart. (**C**) Percent of Tat-binding peaks within individual repeat element subtypes is shown for the top 20 enriched elements.

To determine whether Tat binding regions are associated with specific genomic features, we obtained the coordinates of various DNA elements from the UCSC table browser website [12]. To our surprise, we found that 53% of Tat bound regions lie within repeat-masker (rmsk) regions, which record repeat elements found by RepeatMasker [13], [14] (Figure 2A). We systematically determined the enrichment of Tat binding in various repeat elements and found that, strikingly, 58 percent of all repeat elements bound by Tat are Alu repeats (Figure 2B). In fact, the top ten repeat element types bound by Tat all belong to the Alu family of DNA repeats (Figure 2C). These data reveal that a large fraction of Tat binding regions in the genome are within DNA repeat elements, especially the Alu sequences.

### Tat binding is enriched in the middle to the 3′ end of Alu elements

**Figure 3:**
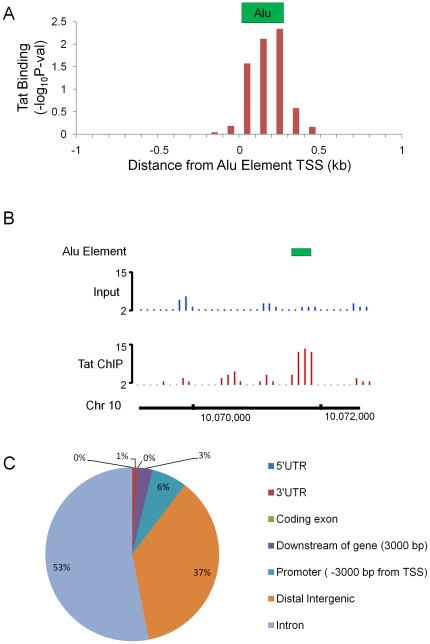
Analysis of Tat binding to Alu elements. (**A**) Average *P*-value of Tat-binding profile centered at the start of Alu elements for all Tat-bound Alu elements is shown. The x axis depicts ±1 kb away from start of Tat-bound Alu elements in 100-bp intervals. The y axis denotes −log_10_ of Tat-binding enrichment *P*-value of ChIPed Tat DNA count over input DNA count. (**B**) Genome browser representation of Tat enrichment profile compared to the input DNA at a representative Alu element is shown. The peak height corresponds to read counts. (**C**) Distribution of Tat-bound Alu elements with respect to gene structure is shown as a pie chart.

Since more than one third of Tat-bound genomic regions contain Alu elements, we sought to determine the binding profile of Tat to Alu elements. As shown in Figure 3A, Alu elements have an average size of 300 base pairs (bp). We generated an average profile of Tat binding centered at the start of all Tat-bound Alu elements. The profile of Tat enrichment over Alu elements was generated by averaging *P*-values of Tat binding enrichment in 100-bp windows ±1 kb from the start of Alu elements. Tat binding on average peaked in the middle of Alu elements with a skewed enrichment toward the 3′ end and downstream regions, up to 200 bp past the average length of an Alu element (Figure 3A). Figure 3B shows an example of Tat binding at an Alu element in an intergenic region of chromosome 10 as indicated. We then asked whether Tat-bound Alu-elements are enriched at specific genomic regions using the CEAS software [11]. The Tat-bound Alu elements are primarily located in introns and intergenic regions of the genome (Figure 3C). In comparison to global Tat binding patterns, Alu elements bound by Tat are more enriched within introns (45% vs 53%). Essentially none of the Tat-bound Alu elements was located within coding exons. Altogether, our data reveals that Tat binds specifically to a fraction of Alu elements within the human genome. These Alu elements may be near or within the introns of genes with functions potentially related to the viral life cycle such as the Alu element shown in Figure 3B.

### Genomic regions bound by Tat are associated with genes enriched in T cell-related functions

**Figure 4:**
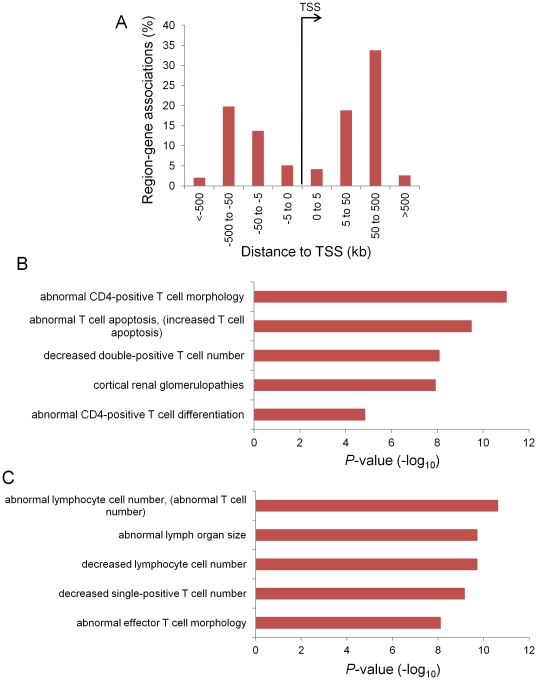
Functional annotation of Tat binding regions. (**A**) The bar chart shows the distribution of the distances between Tat binding peaks and transcriptional start sites (TSS). Note that a Tat peak may be assigned to more than one gene. Functional annotation of (**B**) Tat-bound regions and (**C**) Tat-bound Alu elements, using gene ontology terms generated by Mouse Genome Informatics (MGI) from mouse homologous gene knock out phenotypes. The x-axis values are −log_10_ of binomial raw *P*-values.

As the majority of genomic regions bound by Tat are at least 3 kb away from any TSS, we asked if the nearest genes to Tat binding sites are enriched for specific functions. To observe long range interactions between Tat binding sites and their target genes, we used the Genomic Regions Enrichment Annotations Tool (GREAT) [15] to determine whether genomic regions bound by Tat are located in potential *cis*-regulatory regions of genes important for HIV function. GREAT analysis of Tat-bound regions revealed that the genes potentially associated with distal Tat binding are mostly 5 kb or further away from Tat binding sites. This distribution implies that there may be long range interactions between Tat and its potential target genes (Figure 4A). In support of this, genes associated with regions bound by Tat are significantly enriched in Mouse Genome Informatics (MGI) Phenotype ontology terms related to T cell function (Figures 4B and 4C). MGI analyzes the knockout phenotypes of mouse genes that are homologous to the queried human genes. The mouse homologues of the potentially Tat-regulated genes exhibit knockout phenotypes such as changes in T cell morphology and reduced number of CD4+ and CD8+ T cells (Figure 4B). Similar gene ontology terms were observed when only genes associated with Tat-bound Alu elements were used for MGI analysis (Figure 4C). These data suggest that Tat may exert its effects on the host genes by binding to distant *cis*-regulatory elements.

### Global gene expression changes in Jurkat-Tat cells

**Figure 5:**
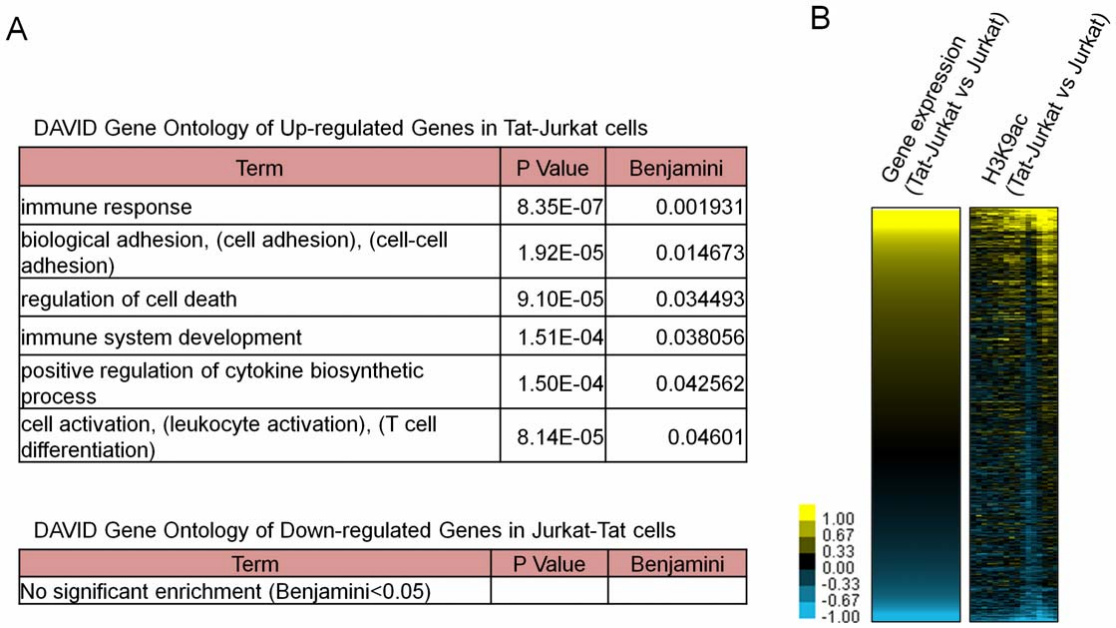
Gene expression profile of Jurkat-Tat cells. (**A**) Gene ontology annotation of genes with more than two-fold change in expression using DAVID. (**B**) Relationships of gene expression and H3K9ac changes to Tat-binding are shown. Genes are arranged in descending order based on their expression in Jurkat-Tat versus Jurkat cells. Each row represents one gene. For H3K9ac ChIP-chip analysis, each column represents a 500-bp window, spanning −5.5 to +2.5 kb of annotated TSS. Moving average of H3K9ac enrichment in three consecutive 500-bp windows is shown in each column (right panel).

To relate global gene expression changes to Tat binding, we used Agilent microarrays to compare global gene expression between Jurkat and Jurkat-Tat cells. Overall, 475 and 319 transcripts showed greater than two-fold increase and decrease in gene expression, respectively. To investigate the functions of the deregulated genes and their relevance to Tat over expression, we performed Gene Ontology (GO) enrichment analysis using DAVID [16]. Genes overexpressed in Jurkat-Tat cells are enriched in cell immune response, cell adhesion, and regulation of cell death (Figure 5A). However, for the down-regulated genes, no significant GO enrichment was found. To determine whether Tat binding is associated with increased or decreased expression of its target genes, we used the GREAT assigned gene-peak associations to correlate Tat binding to gene expression. Surprisingly, we found no significant correlation. Additionally, we performed ChIP combined with Agilent promoter microarrays (ChIP-chip) to monitor changes in histone H3 lysine 9 acetylation (H3K9ac)—a histone modification associated with gene activity—in Jurkat-Tat versus Jurkat cells. We found that changes in H3K9ac correlated positively with gene expression changes; promoter regions of gene that are up- and down-regulated in Jurkat-Tat cells have higher and lower levels of H3K9ac, respectively, compared to Jurkat cells (Figure 5B). These data indicate that changes in gene expression are associated with similar changes in histone acetylation but Tat may have more subtle effects on gene expression that is not detected by our microarray analysis.

### Tat in Jurkat-Tat cells binds to locations occupied by CBP and ETS1

To determine if Tat binding sites are associated with specific chromatin marks, cellular transcription factors or co-factors, we searched the Gene Expression Omnibus (GEO)[17] for published ChIP-seq data in Jurkat or Jurkat-Tat cells. We found five datasets that examined global distributions of ETS1, CBP, RUNX1, H3K4 tri-methylation (H3K4me3) and H3K27me3 in Jurkat cells (GSE23080, GSE17954 [18]). No published dataset in Jurkat-Tat cells was found. ETS1 is a transcription factor that is highly expressed in lymphoid lineage cells and is important for regulating functions of immune cells [19]. In mouse models, inactivation of ETS1 leads to T cell apoptosis [20]. ETS1 and Tat were previously shown to bind at the same region upstream of IL-10 promoter and induce IL-10 transcription [21]. P300 and CBP HATs acetylate Tat and serve as co-activators of Tat-dependent HIV-1 gene expression [3]. RUNX1 is a member of Runt-related transcription factor (RUNX) family of genes that function in normal hematopoiesis. H3K4me3 and H3K27me3 are histone modifications that are generally associated with gene activity and repression, respectively [22].

**Figure 6:**
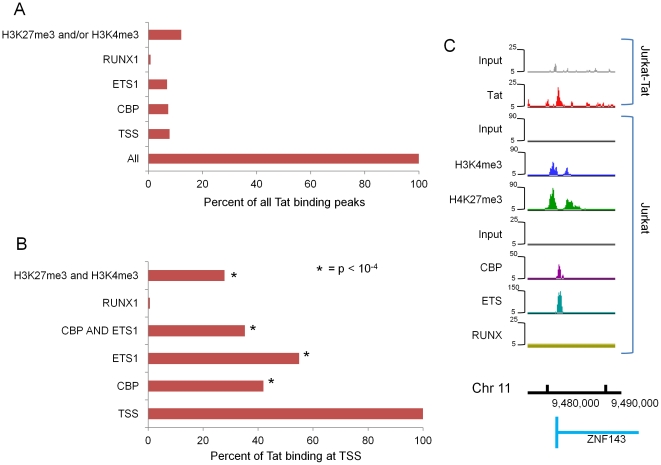
Tat binding peaks in Jurkat-Tat cells are associated with specific cellular factors and chromatin marks in Jurkat cells. (**A**) Percent of all Tat peaks in Jurkat-Tat cells that are bound by CBP, ETS1, RUNX1, H3K4me3 and/or H3K27me3 in Jurkat cells is shown. (**B**) Percent of Tat peaks at TSS in Jurkat-Tat cells that are bound by CBP, ETS1, RUNX1, H3K4me3 and H3K27me3 in Jurkat cells is shown. (**C**) Shown is the Tat enrichment profile at the ZNF143 gene promoter in Jurkat-Tat cells compared to CBP, ETS1, RUNX1, H3K4me3, H3K27me3 enrichment at the same genomic location in Jurkat cells. The peak heights (y-axis) correspond to read counts. The x-axis represents the genomic coordinates.

We downloaded ChIP-seq raw data from GEO [17] and compared each ChIP channel to its corresponding input DNA from the same experimental set. Using the same peak finding algorithm described above with a *P*-value cutoff of <10^−4^, we defined significant peaks of CBP, ETS1, H3K4me3, H3K27me3 in Jurkat T cells. For RUNX1, we found much less significant reads using *P*-value of 10^−4^. To get more similar numbers of total significant peaks, *P*-value of 10^−2^ was used to determine significant reads for RUNX1. Significant peaks found in Jurkat T cells were then compared to Tat binding sites in Jurkat-Tat cells. We defined positive co-occupancy of each factor with Tat when there was at least one significant peak of binding within ±500 bp of the Tat-binding sites. Figure 6A shows the fraction of all (2074) Tat binding sites that are occupied by the indicated factors in Jurkat cells. Only ∼12% of all Tat binding sites coincide with H3K4me3 and/or H3K27me3 and even less so with CBP or ETS1. However, when the analysis is limited to the Tat binding sites within ±1 kb of TSS regions (162 sites), 55% and 42% of Tat peaks are bound by ETS1 and CBP, respectively, in Jurkat cells; 35% are co-occupied by both factors (Figure 6B). Only 1% of Tat binding sites near TSS overlap with RUNX1 binding in Jurkat cells with *P*-value of 10^−2^ and 10^−4^. Furthermore, 28% of Tat TSS binding sites are enriched for H3K4me3 and H3K27me3 in Jurkat cells, including 58% of binding sites with both ETS1 and CBP bound near the TSS (Figure 6B). Figure 6C shows an example of such genes. The promoter of ZNF143 gene is co-occupied by ETS1 and CBP and enriched for H3K4me3 and H3K27me3 in Jurkat cells. The same region is bound by Tat in Jurkat-Tat cells. In contrast, no significant co-occupancy of Tat in Jurkat-Tat and RUNX1 in Jurkat cells was found near TSS. Although the occupancy of these factors in Jurkat-Tat cells is not known, our analysis raises the possibility that specific cellular transcription factors may help recruit Tat or denote the genomic regions to which Tat binds.

## Discussion

We have generated a global map of Tat binding to the human genome. Surprisingly, the majority of Tat target regions lie within DNA repeat elements. In fact, over 30% of all Tat target regions are located at or near Alu elements. Interestingly, Tat increases the transcription of Alu repeat elements by increasing the activity of cellular transcription factor TFIIIC in Jurkat cells [23]. Since Alu elements antagonize the interferon-induced protein kinase R (PKR) activation [24] and PKR is known to repress protein synthesis when cells are under stress, Tat binding at Alu elements may be important to enable efficient viral replication in the host cell. In addition, these Alu elements may affect regulation of genes with functions related to HIV-1 biology.

In relation to gene regulation, Tat binding sites are distal to genes with functions in T cell biology as determined by knockout models in mice. These data suggest that Tat may exert its effects on its target genes through distant regulatory elements. If so, then Tat binding, in and of itself, may highlight previously unknown *cis*-regulatory elements within the genome. We did not find a significant correlation between Tat binding and gene expression. The effects of Tat on gene expression may be too subtle for the microarrays to detect. Alternatively, Tat may affect the regulation of its target genes through effects on mRNA structure that is not evident by expression analysis.

We found only a few genes with Tat binding to the vicinity of their TSS. This is partly due to the stringent cutoff used in this study to maintain a False Discovery Rate (FDR) under 5%. For instance, gene promoters previously shown to be bound by Tat binding such as IL-6 [4] and ß2 microglobulin [6], showed some Tat binding above background but did not pass our criteria for statistical significance. Also, previous studies used different cell culture systems to determine Tat binding in vivo. These differences may also lead to identification of different sets of target genes.

Since Tat is not known to bind DNA directly, the mechanism by which Tat binds to specific regions of the genome may partly involve interactions with host cellular factors. By comparing our Tat binding data to the published datasets in Jurkat cells, we found that Tat binds to gene promoters that were bound by ETS1 and CBP, but not with RUNX1, in non-Tat expressing Jurkat cells. A similar positive relationship was found between Tat binding and two histone methylation marks, H3K4me3 and H3K27me3. These data raise the possibility that Tat may distinguish its target regions through specific host transcription co-factors or chromatin marks. However, our data does not include or exclude ETS1, CBP or the histone modifications as the mediators of Tat recruitment. Binding analyses of specific Tat mutants that disrupt its interaction with specific cellular factors are required to determine potential mechanisms of Tat recruitment. It is also possible that Tat binds to the genome through an RNA component. Nonetheless, our results demonstrate that in addition to known roles for Tat in enhancing elongation of viral transcription, Tat also binds to the host genome at specific genomic locations with potentially important consequences for the viral life cycle.

## Materials and Methods

### Cell culture

Jurkat T cells were obtained from ATCC (TIB-15) and cultured under standard tissue culture conditions. Jurkat-Tat cells were obtained from the NIH AIDS Research & Reference Reagent Program and maintained in RPMI supplemented with 10% fetal bovine serum, 1% penicillin and streptomycin and 800 µg/ml of G418.

### Western blotting

Cells were lysed with RIPA buffer (50 mM Tris-HCl pH 8.0, 150 mM NaCl, 1% NP-40, 0.1% SDS, 1% sodium deoxycholate) supplemented with protease inhibitors (Roche). Cell lysates were subjected to SDS-PAGE and analyzed by Western blot using standard procedures. The antibodies used for Western blotting were as follows: Anti-Tat (Abcam ab43014), Anti-Beta-actin (Abcam ab8224), Anti-H3 (Abcam ab10799).

### Chromatin immunoprecipitation and ChIP-seq library preparation

Jurkat-Tat cells in exponential growth phase were fixed with 1% formaldehyde (v/v) for 10 min at 37°C. Fixation was stopped by addition of glycine to a final concentration of 140 mM. Cell were lysed and chromatin was digested with the micrococcal nuclease from *S. aureus* (Roche) for 90 min at 4°C according to the manufacturer's instructions, re-suspended in lysis buffer and sonicated with Misonix ultrasonic liquid processor. 1% of the lysate was used as an input control. Lysates were immunoprecipitated with 5 µg of anti-Tat antibodies (Abcam ab43014) using the standard ChIP protocol. Both purified input and Tat chromatin samples were used to prepare ChIP-seq libraries according to the manufacturer's instructions (Illumina). Libraries were sequenced using Illumina Genome Analyser II to obtain 76 bp-long reads.

### Chromatin immunoprecipitation and microarray hybridization

Jurkat-Tat and Jurkat cells in exponential growth phase were fixed with 1% formaldehyde (v/v) for 10 min at 37°C. Fixation was stopped by addition of glycine to a final concentration of 140 mM. Histone H3 acetyl lysine 9 antibody (Upstate 07-352) was used for ChIP. The ChIP-chip and subsequent analysis were performed essentially as described previously [25].

### ChIP-seq data analysis

Sequenced reads were base-called using the standard Illumina software. Bowtie 0.12.7 was used to align the reads to the Human genome (Hg19) allowing up to two mismatches; reads that aligned to more than one location in the genome were discarded. For unaligned reads, 5 bp from the 5′ end and 25 bp from the 3′ end of the reads were trimmed and re-aligned to the genome. The total number of reads in the input sample was normalized to the ChIP counts. Genomic regions with significant enrichment of Tat chromatin over input chromatin were calculated within 100 bp windows that tiled the genome. The *P*-value for enrichment of ChIP versus input reads was calculated using the cumulative Poisson distribution.

### Expression profiling

Total RNA was isolated from Jurkat and Jurkat-Tat cells using the RNeasy Mini kit (Qiagen). cRNAs were generated from 250 ng of total RNA and labeled with Cy3 (Jurkat) or Cy5 (Jurkat-Tat) using the Low Input Quick Amp Labeling Kit (Agilent) according to manufacturer's instructions. Labeled cRNAs were hybridized to the Agilent Human whole-genome array (G2534-600110) according to Agilent protocol. Raw intensity data from resulting gene expression data were normalized by medium background-subtracted intensities between Cy5 and Cy3 channels followed by LOWESS normalization using Matlab. Normalized log_2_ ratios of Jurkat-Tat cRNAs (Cy5) over Jurkat cRNAs (Cy3) were calculated and results from replicates were averaged. Gene ontology enrichment analysis was performed using The Database for Annotation, Visualization and Integrated Discovery (DAVID) v6.7 [16].

All data have been deposited in the Gene Expression Omnibus (GEO) [17] under accession number GSE30739.

## Supporting Information

Figure S1 **Tat is expressed in Jurkat-Tat cells.** Western blots of the indicated factors in normal Jurkat T cells (Jurkat) and Jurkat cells stably expressing Tat (Jurkat-Tat).
(TIF)
