# Supplementary figures and images for: Genome-Wide Binding Map of the HIV-1 Tat Protein to the Human Genome
Source: PLoS One. 2011 Nov 4;6(11):e26894. doi: 10.1371/journal.pone.0026894 (PMC3208564; doi:10.1371/journal.pone.0026894)

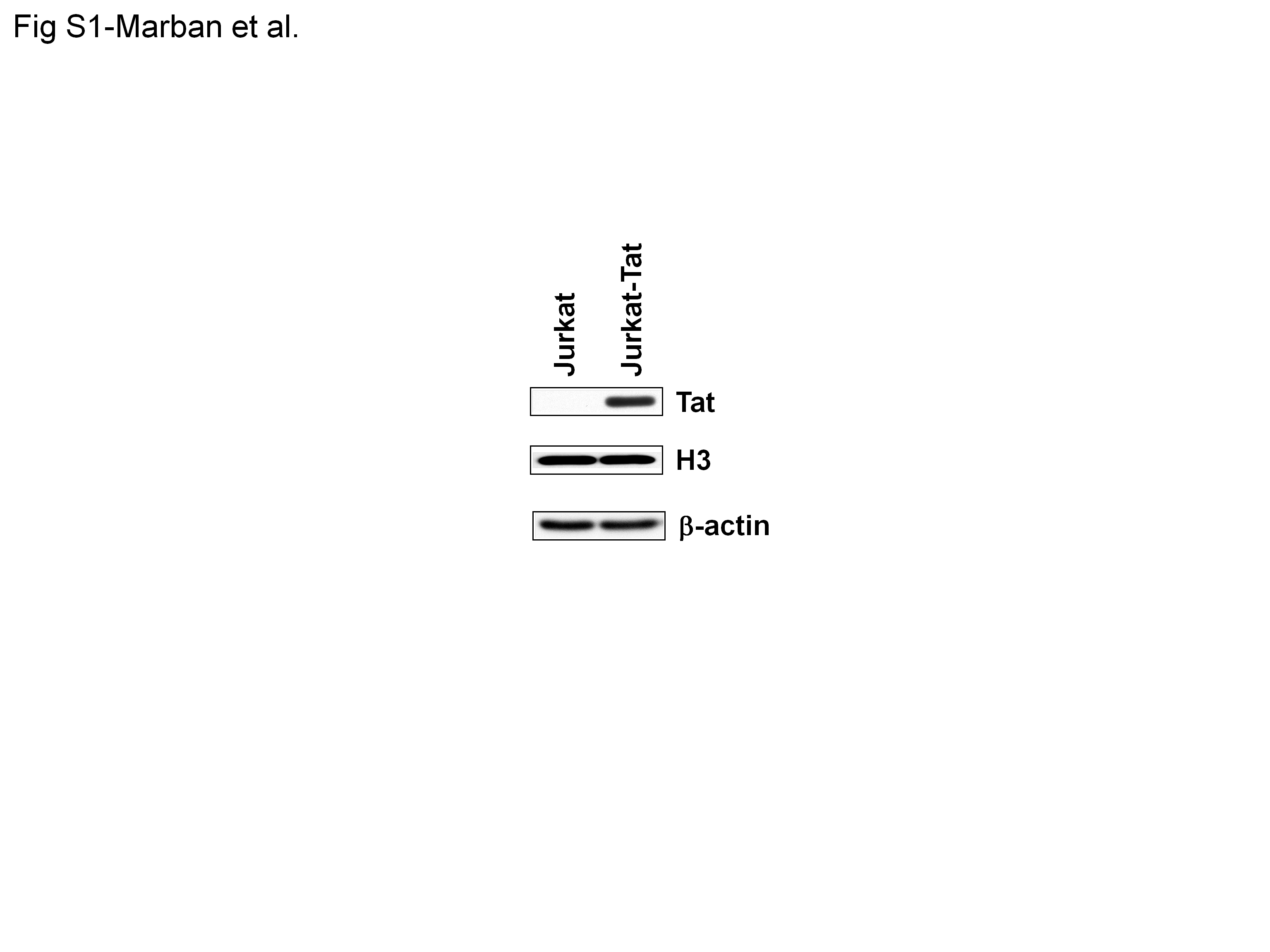

Supplement: Figure S1 — Tat is expressed in Jurkat-Tat cells. Western blots of the indicated factors in normal Jurkat T cells (Jurkat) and Jurkat cells stably expressing Tat (Jurkat-Tat). (TIF) [file pone.0026894.s001.tif]
